# Three-dimensional printed poly (L-lactide) and hydroxyapatite composite for reconstruction of critical bone defect in rabbits

**DOI:** 10.1590/ACB360404

**Published:** 2021-05-21

**Authors:** Bruno Watanabe Minto, Arícia Gomes Sprada, José Aloizio Gonçalves, Brenda Mendonça de Alcântara, Thiago André Salvitti de Sá Rocha, Ana Carolina Valentim Hespanha, Carolina Quarterone, Maressa da Rocha Sartori, Alessandre Hataka, Ricardo Andres Ramirez Uscategui, Luis Gustavo Gosuen Gonçalves Dias

**Affiliations:** 1 Assistant Professor. Universidade Estadual Paulista “Júlio de Mesquita Filho” – Faculty of Agrarian and Veterinary Sciences – Department of Clinical and Veterinary Surgery – Jaboticabal (SP), Brazil.; 2 Assistant Professor. Centro Universitário de Maringá – Department of Veterinary Anatomy – Maringá (PR), Brazil.; 3 Fellow Master degree. Universidade Estadual Paulista “Júlio de Mesquita Filho” – Faculty of Agrarian and Veterinary Sciences – Graduate Program in Veterinary Surgery – Jaboticabal (SP), Brazil.; 4 Fellow PhD degree. Universidade Estadual Paulista “Júlio de Mesquita Filho” – Faculty of Agrarian and Veterinary Sciences – Graduate Program in Veterinary Surgery – Jaboticabal (SP), Brazil.; 5 Assistant Professor. Universidade Brasil – Department of Clinical and Veterinary Surgery – Descalvado (SP), Brazil.; 6 Assistant Professor. Centro Universitário de Maringá – Department of Veterinary Surgery – Maringá (PR), Brazil; 7 Graduate student. Centro Universitário de Maringá – Maringá (PR), Brazil; 8 Assistant Professor. Universidade Estadual Paulista “Júlio de Mesquita Filho” – Faculty of Agrarian and Veterinary Sciences – Department of Veterinary Clinical Sciences – Botucatu (SP), Brazil.; 9 Associate Professor. Universidade Federal dos Vales do Jequitinhonha e Mucuri – Institute of Sciences – Department of Veterinary Clinical Sciences – Unaí (MG), Brazil.

**Keywords:** Biocompatible Materials, Osteogenesis, Tissue Scaffolds, Tissue Engineering, Rabbits

## Abstract

**Purpose:**

To use a 3D printed poly (L-lactide) acid (PLLA) and hydroxyapatite (HA) composite as a bone substitute for reconstruction of a critical bone defect in the radius of rabbits.

**Methods:**

A 1.5 cm ostectomy was performed in the radial diaphysis of 60 New Zealand white rabbits. The rabbits were divided into three groups according to surgical treatment of the bone defect (group I – control, group II – bone graft, group III – 3D PLLA). Each group was divided into four subgroups with different radiographic and histopathologic evaluation times (T1 – 15 days, T2 – 30 days, T3 – 60 days, T4 – 90 days).

**Results:**

The implant group had greater clinically lameness (p = 0.02), edema (p = 0.007), pain (p = 0.04) and more complications at the surgical site (p = 0.03). Histologically, this group showed greater congestion (p = 0.04), hemorrhage (p = 0.04) and inflammation. Osteogenesis was microscopically similar between days (p = 0.54) and treatments (p = 0.17), even though radiographically, more effective bone healing occurred in the graft group (II), with more callus and bone bridge formation.

**Conclusions:**

The customization of a 3D PLLA/HA scaffold was successful. However, in animals receiving the polymer-ceramic composite less bone callus and bone bridge was formed compared to the graft group.

## Introduction

A bone defect, which is not expected to consolidate without surgical or complementary intervention, is defined as a critically sized defect^1^. Such defects are typically associated with high energy trauma, open fractures, infections and resection of bone tumors. Avascular nonunion, especially when associated with osteomyelitis, vascular injuries and inadequate stabilization can create challenging repair scenarios^2^,^3^.

Despite developments in bone tissue engineering, the treatment of critical sized defects has remained challenging, and complications have a significant economic impact^4^. Autologous bone graft has been the gold standard for treatment of bone defects. However, its use is hampered by donor site morbidity and limited available bone volume. Bone tissue engineering has recently offered a real alternative to autologous bone graft. Biomaterials and manufacturing methods, including three-dimensional (3D) printing, have emerged to fabricate scaffolds to assist bone repair^5^–^7^.

Three-dimensional printing has several applications in medicine, such as surgical planning tools, anatomical studies and creation of prostheses^8^. More recently, 3D manufacturing based on rapid prototyping has aided the treatment of challenging diseases and pathological conditions. Additionally, this technology allows the creation of customized composites to replace patient-specific bone segments^9^,^10^.

The purpose of this study was to develop a bone substitute using 3D printer technology and to implant it in critical radial defects in rabbits. Clinical, radiographic and histologic evaluations were performed in a comparative study using iliac crest autografts. The hypothesis was that the 3D printed scaffold would successfully fill the bone gap and allow bone healing.

## Methods

Ethical approval was obtained from the institutional ethical committee (protocol No. 9417/15). Sixty female, skeletally mature (> 7 months) rabbits (O*ryctolagus cuniculus*), weighing between 4 and 5.5 kg were used. The animals were divided into three groups (control, graft and poly [L-lactide] acid [PLLA]) according to the surgical treatment of the bone defect (created by a 1.5 cm ostectomy in the right radius diaphysis): control was composed of 20 animals without any grafting; in animals in the graft group, the bone defect was filled with an iliac crest autologous graft; finally, animals in PLLA received a 3D printed bone implant for reconstruction of the bone defect. All groups were divided into four subgroups according to the radiographic and histopathologic evaluation times: 15 (T1), 30 (T2), 60 (T3) and 90 days (T4) postoperatively.

### Three-dimensional scaffold preparation

**Figure 1:**
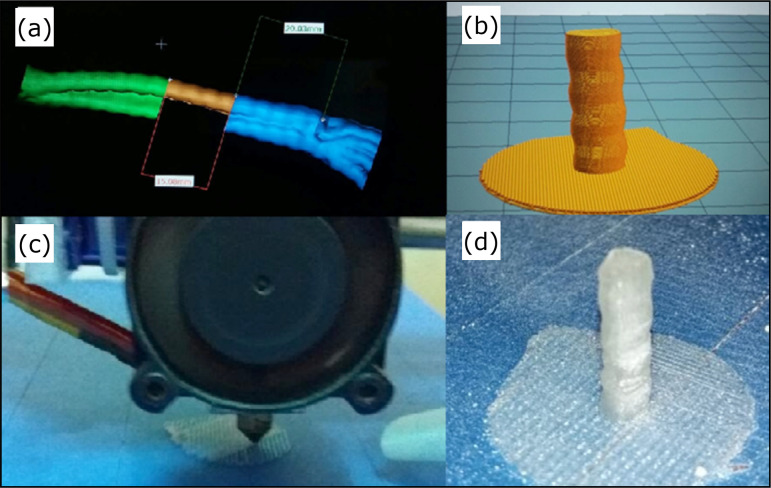
3D printing process. **(a)** Computed tomography image; (b) Three-dimensional images of the forearm in InVesalius software for file conversion in DICOM to STL format; **(c)** Three-dimensional PLLA and HA composite printing process; **(d)** Final PLLA and HA composite after 3D printing.

Through sequential computed tomography (GE Speed Helical – Chicago – IL – USA) with 120 kV, 130 mA and 1 mm slice thickness, images of the right limb of all animals in group III were obtained. These digital imaging and communications in medicine (DICOM) images were reconstructed in three-dimensions and converted into STL format by using InVesalius software, allowing manipulation of the images. Segment cuts and separation of the radius from the ulna were made with blender software (Meshmixer version 3.5.474 – Autodesk Inc), delimiting the area of interest for printing, which was a 1.5 cm segment of the radius 2 cm above the radiocarpal joint (Fig. 1). This virtual replica was printed with direct drive extrusion (Original Prusa i3). The material used was the composite of PLLA, an absorbable filament and hydroxyapatite powder. The implants were sterilized with ethylene oxide at the end of the process.

### Surgical procedures

Preanesthetic medication consisted of ketamine hydrochloride 20 mg·kg^–1^ (Cetamin), midazolam maleate 2 mg·kg^–1^ (Dormonid) and morphine sulphate 2 mg·kg^–1^ (Dimorf) intramuscularly (IM). General anesthesia was induced and maintained with isoflurane (Isoforine) vaporized in 100% oxygen with the use of an inhalation mask and spontaneous respiration. A right brachial plexus block was performed in all animals and sacroiliac regional anesthesia was given to animals in graft group using lidocaine 6 mg·kg^–1^ 2% (Lidovet) without vasoconstrictor.

With the animal in right lateral recumbency, a 3-cm longitudinal skin incision was made on the dorsomedial face of the right limb. Subcutaneous tissue and musculature were retracted to expose the diaphysis of the radius and the periosteum was removed by blunt dissection. The ostectomy was performed 2.0 cm above the carpus joint, removing a 1.5-cm segmental defect with the aid of an oscillating saw. Care was needed during surgery due to the proximity of the radius to the ulna. For bone fragment removal, the interosseous ligament was incised. Following osteotomy, treatment was conducted according to the animal group.

**Figure 2:**
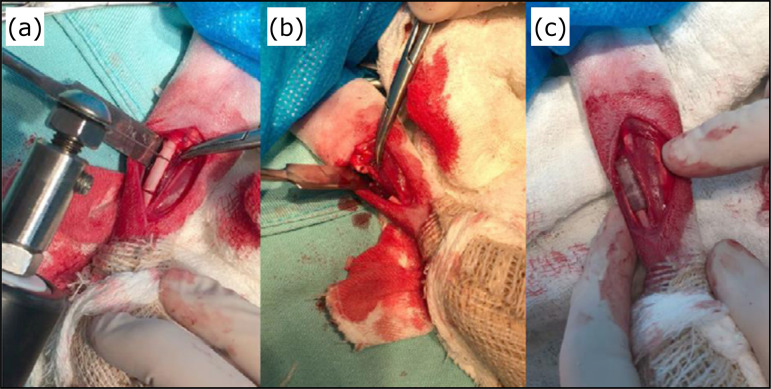
Surgical procedure of the implant group using a 3D printed bone substitute for treatment of a critical defect in the radius diaphysis of a New Zealand rabbit. **(a)** After radius exposure, two osteotomies were performed with oscillatory saw, the distal cut was made 2 cm above the radiocarpal joint; **(b)** Removal of the radius segment of 1.5 cm, creating the critical bone defect; **(c)** 3D bone substitute was implanted into the critical bone defect.

In control, the bone defect was left empty and subcutaneous tissue and skin were sutured in routinely fashion. In group II, a skin incision was made on the craniodorsal aspect of the ilium crest. Lateral and medial musculature were removed, exposing the bone of the ilium. Using an oscillatory saw, a segment of corticocancellous graft was harvested and immediately implanted in the radial defect. In the animals of group III, the 3D printed bone substitute was placed into the defect so that its extremities remained in close contact with the bone edges (Fig. 2).

Postoperative medication consisted of dipyrone25 mg·kg^–1^ (D-500) subcutaneously (SC) twice a day (BID), tramadol hydrochloride 4 mg·kg^–1^ (Tramal) BID SC, meloxicam 0.1 mg·kg^–1^ (Maxicam) once a day (SID) SC, all for three days and enrofloxacin 5 mg·kg^–1^ (Zelotril) BID, SC for five days. The animals were clinically assessed for ambulation, limb support, presence of pain and inflammation in the affected limb. The observation was always performed by the same observer on the 7th, 15th, 30th, 60th and 90th day after surgery, according to each group and subgroup, following the classification of Stasiak^11^.

### Radiographic analysis

**Figure 3:**
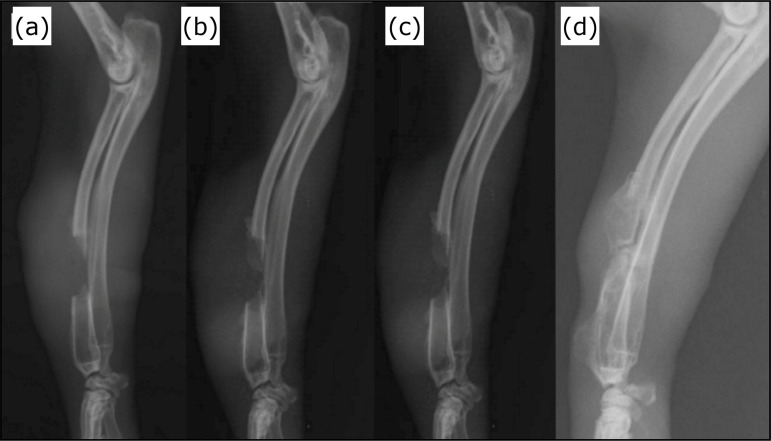
Midlateral postoperative radiographs of the right thoracic limb of New Zealand rabbit number 58, implant group, from the study. (a) 15 days postoperative; (b) 30 days postoperative; (c) 60 days postoperative; (d) 90 days postoperative.

Craniocaudal and mediolateral radiographs (100 mA, 70 kV) were taken in the immediate postoperative period, and 15, 30, 60 and 90 days after surgery, depending on the subgroup (T1, T2, T3 and T4, respectively) (Fig. 3). Radiographs were analyzed by three evaluators blinded in relation to the groups. The images were assessed for periosteal reaction, bone callus volume and bone bridge quality, receiving scores from 1 to 4, as described by Öztürk *et al*.^12^.

### Histopathologic analyses

For histopathologic analysis, the radius and ulna of each experimental group were collected and dissected after euthanasia. Samples were fixed in 10% buffered formaldehyde for four days, then washed overnight in running water to remove excess formaldehyde. The samples were decalcified in 10% nitric acid solution for three to five days, after which they were treated with 5% sodium sulphate solution for 24 h. Subsequently, the samples were dehydrated in alcohol 70 and 80% and absolute for 60 min each. Then, immediately diaphanized in absolute xylene for 50 min. Finally, the samples were set in histological paraffin for 60 min. From the blocks, four micrometer histological sections were made using a semiautomatic microtome (LEICA RM 2155 – rotatory microtome). The histologic slides were stained by Masson’s hematoxylin and eosin and trichrome techniques. Examinations were performed using a light microscope to compare fibrous tissue, cartilaginous and osteoid tissue neoformation during the bone regeneration process. The evaluations were classified using scores(1 to 4) based on the presence of changes, where one is absence of change, two discrete, three moderate and four marked changes. The characteristics analyzed were the presence of congestion, hemorrhage, inflammatory infiltrate and collagen characterization. The evaluation was performed by a single experienced evaluator.

### Statistical analysis

Statistical analysis was performed with software R(R Foundation for Statistical computing, Vienna, Austria). Radiographic evaluations were compared among the observers by the Bland–Altman concordance test. Clinical, radiographic and histopathological parameters were subsequently compared between the treatment groups, the days of evaluation and the interaction of these factors by the Friedman test and Dunn’s post-test, presenting their results as mean ± IQR (interquartile range). Significance was set for all tests at 5% (p < 0.05).

## Results

All animals used the operated limb soon after anesthetic recovery and, during the entire experimental period, no animal had severe lameness, as discussed below.

**Figure 4:**
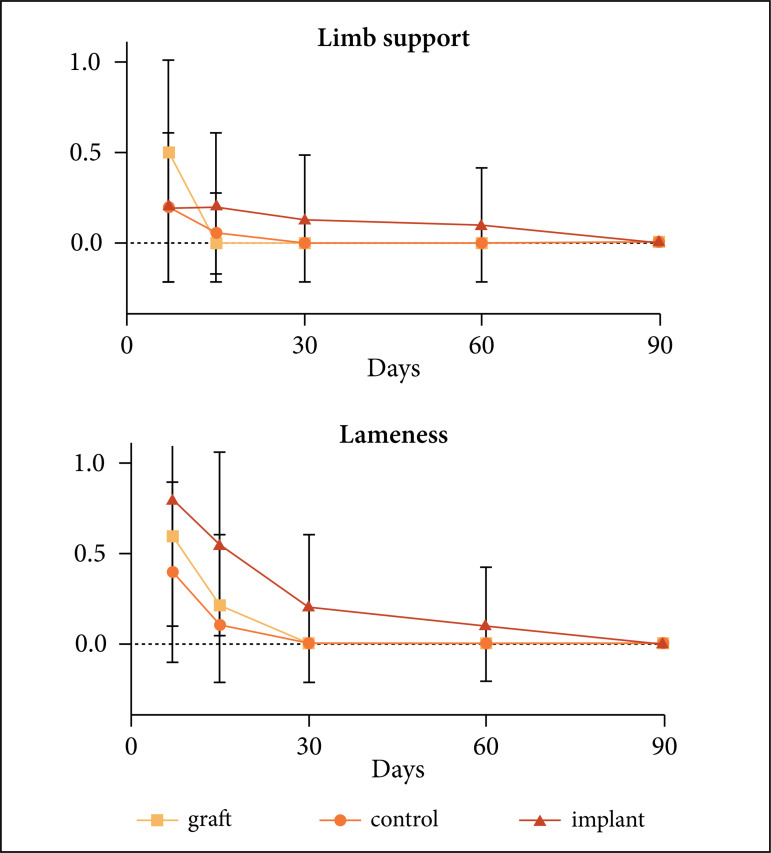
Graph showing limb support and lameness after segmental ostectomy of the radio in rabbits according to the treatment during the postoperative evaluation periods of 7, 15, 30, 60 and 90 days.

Weight-bearing was similar between groups (p = 0.1954), increasing significantly after the 15th day of evaluation in all groups (p = 0.0443). Lameness was greater (p = 0.0243) in the implant group when compared with other groups on day 7 and 30 and decreased gradually (0.0225) with time (Fig. 4).

**Figure 5:**
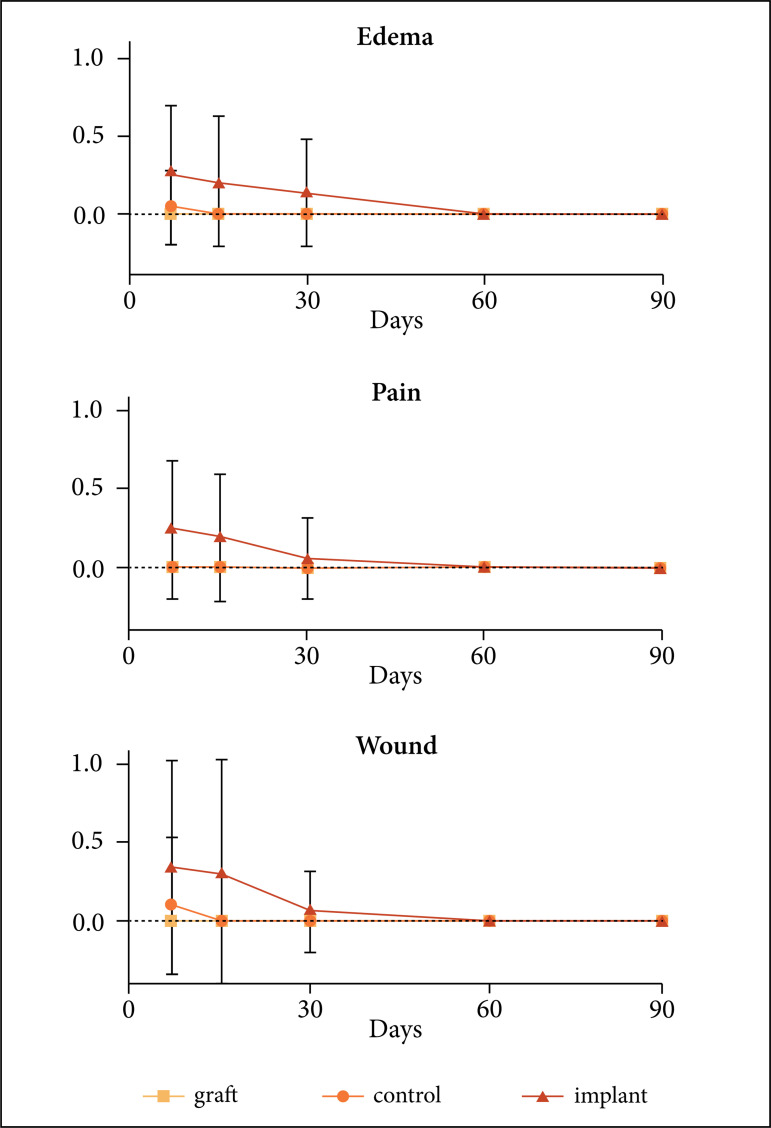
Graph showing presence of edema, pain and wound complication after segmental ostectomy of the radio in rabbits according to the treatment during the postoperative evaluation periods of 7, 15, 30, 60 and 90 days.

Edema was greater (p = 0.0074) in the implant group than in others from 7 to 30 days of evaluation, and there was no influence of time (p = 0.1496). Pain was greater (p = 0. 0497) in the implant group at the 7th and 15th days and there was no change over time (p = 0.4060). The presence of complications in the surgical wound was greater (p = 0.0308) in the implant group at the 7th and 15th days and there was no influence of time (p = 0.4060) (Fig. 5).

**Figure 6:**
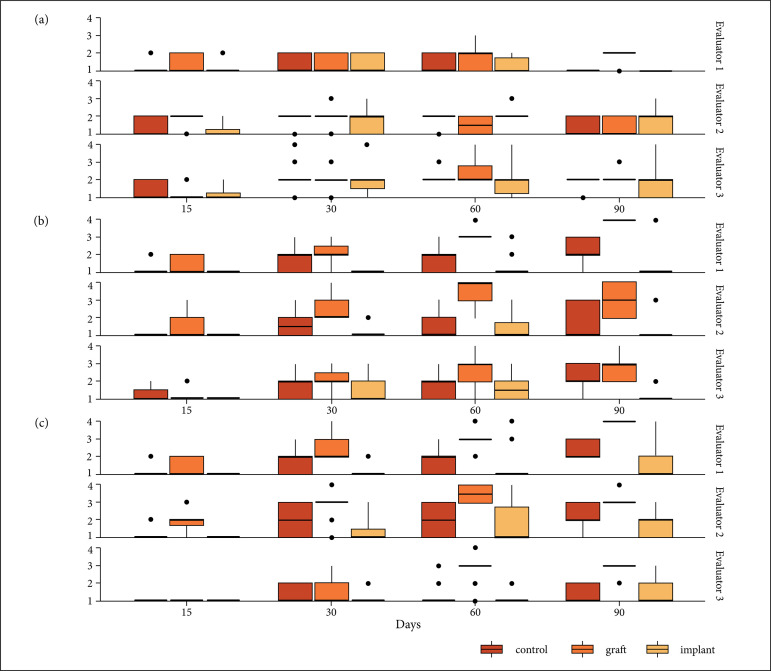
Radiographic results at different periods in the three different groups by the three evaluators. (a) Periosteal reaction; (b) Bone callus volume; (c) Bone bridge quality.

In the radiographic evaluation, evaluator 3 underestimated (p = 0.0001) the periosteal reaction with a bias of 26%. Evaluator 2 underestimated bone bridge (p = 0.0001) with a bias of 22%, whereas bone callus evaluation was similar between evaluators(p = 0.5161). The periosteal reaction was less (p = 0.0048) in the control group at day 90. Bone callus formation was smaller(p = 0.0183) in the implant group at days 30, 60 and 90, and greater in the graft group at days 60 and 90. Bone bridge was smaller (p = 0.0421) in the implant group at 30, 60 and 90 days and greater in the graft group at 60 and 90 days (Fig. 6).

Histopathologic study showed bone consolidation in three animals from the graft group T4, five animals of T3 and one animal from T2. Those animals that did not have lameness had exuberant bone callus formation. In the implant group, foreign body giant cells were identified at the interface between the bone and the implant, mainly in the subgroups T4, T3 and T2. Also, in these subgroups there was pseudocapsule formation involving the implant and, in one animal belonging to T4, an abscess was present.

**Figure 7:**
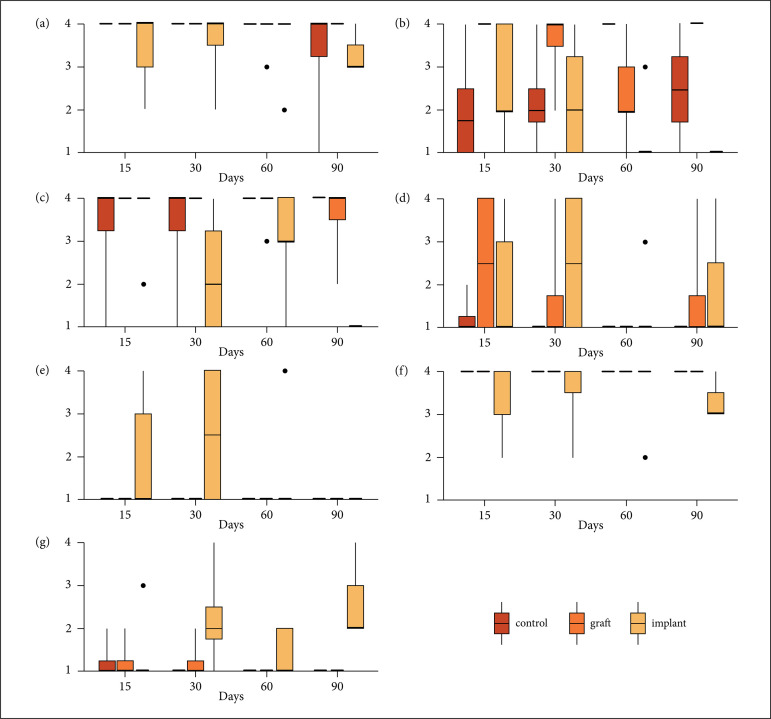
Histological results at different periods in the three different groups. (a) Fibrosis; (b) Chondrogenesis; (c) Osteogenesis; (d) Congestion; (e) Hemorrhage; (f) Collagen; (g) Inflammation.

Histological results were as follows: fibrosis was similar between days (p = 0.4835618) and treatments(p = 0.1353353), as well as chondrogenesis (days p = 0.7185168, treatments p = 0.1737739) and osteogenesis (daysp = 0.5432912, treatments p = 0.1737739). Congestion was similar between days (p = 0.1313505) and greater in the implant group when compared to control (p = 0.04688824). Hemorrhage was similar between days (p = 0.3916252) and greater in the implant group than in the other groups (p = 0.04978707). Collagen was similar between days(p = 0.40300738) and lower in the implant group than in the other groups (p = 0.01831564). Inflammation was similar between days (p = 0.4792326) and greater in the implant group than in the other groups and in the graft group than in the control group (Fig. 7).

## Discussion

In human medicine, treatment of bone defects is challenging due to the great loss of bone tissue and even adjacent tissues. Therefore, the rate of complications is higher in these cases and bone nonunion is a common outcome^4^. The use of grafting and adequate stabilization is essential for a good result in these cases. There are a wide variety of grafts that can be used, including autologous, allogeneic, xenogenic and alloplastic^13^.

The autologous graft is the gold standard for these treatments; however, in some cases there is insufficient material to completely fill the large-scale bone defect and an alternative is the use of biomaterials. In this study, a 3D alloplastic composite made of PLLA and HA, two biocompatible substances that have osteoconductive and osteoinductive properties, was used^14^,^15^. Three-dimensional printing has emerged as a critical tool for bone engineering and allows the repair of large-scale bone defects with optimal patient-specific scaffolds with complex architecture. There are several methods for the creation of 3D compounds, including stereolithography (SLA), digital light processing (DLP), selective laser sintering (SLS), fused deposition modelling (FDM) and others^9^. The 3D structures need specific architecture with porosity that provides an appropriate environment for cell multiplication^16^. In addition, the material must be biocompatible and biodegradable, allowing cell proliferation in their pore network without inflammatory reactions that prejudice tissue repair^17^.

In this study, 3D printing was used to produce the PLA + HA composite. Fused deposition modelling can be used for thermoplastic biomaterials, like PLA, making combination with other biomaterials like HA possible^18^. This process allows the production of 3D structures with complex architecture that can be difficult to achievewith other methods^9^. In the present study, it was feasible and practical to produce a complex 3D composite scaffold with similar anatomy to that of the surgical site. In all animals, the composite produced was anatomical and remainedin the site of application even without the use of implants for fixation, providing load sharing.

Poly (L-lactide) acid has good biocompatibility and biodegradability as a scaffold, permitting cell growth; however, it has mechanical properties that do not assist in load sharing and inflammatory reactions may occur. In combination with HA, a porous composite is formed with a ceramic behavior that improves its mechanical properties, degradation rate and osteoconduction^19^. In this study, the biocompatibility of the scaffold was effective, with no material rejection. In addition, the composite allowed load sharing between the fragments of the radius and maintained its architecture during the study, without deformation even with the compression applied on the biomaterial.

In this study, the signs of inflammation were greater in the animals that received the composite, which showed more edema, pain and lameness. Moreover, giant cells and pseudocapsules were also found involving the biomaterial, suggesting an exacerbated inflammatory reaction. The degradation of scaffolds made of polymers, including PLA, can lead to inflammatory reactions^20^. In addition, rabbits are predisposed to produce exaggerated granulation tissue reactions and the degradation of PLA monomers can lead to a decrease in pH, making cell repair difficult and enhancing the inflammatory process^21^. However, it is reported that there is no significant difference in inflammation caused by the application of HA + PLA in cranial defects in rats^22^.

The gap and movement between the scaffold and the bone fragments are important factors to ensure bone formation, especially in 3D composites^23^. Therefore, although the ulna improves mechanical stability, that allows effective support, the micromovement in the bone defect increases the stress on the composite. Consequently, this micromovement can lead to instability between the scaffold and the bone fragments, resulting in an inflammatory process, reduction of the load sharing and the rupture of cells. Stabilization with a plate, for example, could increase the rigidity of the stabilization and decrease the movement of the composite^5^.

The animals that received the composite had worse clinical and histologic changes when compared to the other groups. However, care is needed in the interpretation between groups II and III, since in the graft group (positive control) the gold standard for bone regeneration was used, which can lead to superior results, as reported by numerous previous studies^10^,^24^. A factor that could assist the scaffold would be the use of precursor cells for osteogenesis to optimize bone healing, since biologically active 3D implants are promising in tissue regeneration^25^.

Additionally, there was less formation of bone callus and bone bridge in group III. However, in the present study, complete degradation of the implant did not occur within 90 days, which may be related to the density of the material obtained from the impression of PLA + HA, increasing rigidity and half-life of the material. Poly (L-lactide) + HA used in maxillofacial surgeries take up to 5 years for complete degradation, maintaining their strength for up to 6 months^26^. Therefore, evaluation for a longer time would be necessary to provide more information about the biodegradability of this composite and, consequently, its capacity to assist bone formation. In addition, this factor may have interfered with the formation of bone callus for up to 90 days, but it does not prevent the formation of bone tissue.

The porosity of the biomaterial influences its ability to house cells and facilitate neovascularization^5^,^10^. In this study, the porosity of the composite was not controlled, and it may have been harmful to osteoconduction. The homogeneity and orientation of the fibers, as well the interaction between PLLA and HA, are important factors that may influence the expected biomechanical and biological performance^27^,^28^. Moreover, pores with a size of 300 µm would be ideal for osteoconduction^29^, although the use of pores with variable sizes from 200 to 400 µm have been shown to have excellent osteoconductive capacity^28^.

## Conclusion

It was possible to create a scaffold with anatomical characteristics similar to the radius in animals in this study. The material had good biocompatibility and allowed cell multiplication around the composite. However, in animals receiving the polymer-ceramic composite, less bone callus and bone bridge was formed compared to the graft group. Factors such as material porosity, mechanical stability and the short evaluation period are limiting factors in the study, and further studies are needed to optimize the use of composite materials for bone tissue engineering.
